# Editorial: Vascular diseases of the brain: insights, progress and lessons learned

**DOI:** 10.3389/fneur.2026.1800781

**Published:** 2026-03-16

**Authors:** Rafael Rehwald, Carole H. Sudre, Pedro L. Vilela, Hans Rolf Jäger

**Affiliations:** 1 Neuroradiological Academic Unit, Department of Translational Neuroscience and Stroke, UCL Queen Square Institute of Neurology, University College London, London, United Kingdom; 2 Unit for Lifelong Health and Ageing at UCL, Institute of Cardiovascular Science, University College London, London, United Kingdom; 3 Department of Computer Science, Hawkes Institute, University College London, London, United Kingdom; 4 School of Biomedical Engineering, King's College, London, United Kingdom; 5 Departamento de Neurorradiologia, Hospital da Luz Lisboa, Lisboa, Portugal; 6 Lysholm Department of Neuroradiology, National Hospital for Neurology and Neurosurgery, London, United Kingdom

**Keywords:** artificial intelligence, cerebral small vessel disease, cerebrovascular disease, neuroimaging biomarkers, neuroradiology, precision medicine, vascular cognitive impairment, vascular imaging

Substantial advances in neuroscience and cerebrovascular research have shaped our understanding of brain health and disease. The convergence of artificial intelligence, increasingly sophisticated neuroimaging techniques, and evolving diagnostic frameworks is reshaping how cerebrovascular diseases are detected, characterized, and managed (1–3). These developments have had tangible clinical impact, contributing to improved outcomes in disorders that remain among the leading causes of death and long-term disability worldwide (4, 5).

Cerebrovascular disease encompasses a heterogeneous group of conditions that can be broadly categorized into pathologies affecting large- and medium-sized cerebral arteries and those involving the small perforating vessels supplying cortical, subcortical, and deep gray matter structures (6). Large-vessel disease includes acute ischemic stroke due to major arterial occlusion, atherosclerosis (including stenosing and vulnerable plaques), dissections, and aneurysmal rupture, entities that have benefited from substantial advances in interventional neuroradiology and clinical management (5–7). This is exemplified by work from Zhang et al., who demonstrate how contemporary endovascular strategies for intracranial aneurysm treatment can achieve favorable safety and efficacy profiles. Complementing these therapeutic advances, emerging imaging biomarkers such as aneurysm wall calcification patterns are improving rupture risk stratification and informing treatment decisions, underscoring ongoing progress in the interventional management of large-vessel cerebrovascular disease (8).

In contrast, cerebral small vessel disease (CSVD) represents a process involving small arteries, arterioles, venules, and capillaries which can have different underlying etiologies, most commonly arteriolosclerosis and cerebral amyloid angiopathy, but also genetic and inflammatory or immunologically mediated forms (9). CSVD accounts for a substantial proportion of ischemic strokes and spontaneous intracerebral hemorrhages and is a major contributor to vascular cognitive impairment and dementia (6).

Although large- and small-vessel disease are often discussed as distinct entities, growing evidence suggests that they are intertwined (10). Shared vascular risk factors—including hypertension, diabetes, hypercholesterolemia, smoking, and aging—drive pathological changes across the cerebrovascular tree (6, 10). Yet the mechanisms linking macrovascular pathology to microvascular injury, and vice versa, remain incompletely understood, though emerging evidence points to shared pathways involving endothelial dysfunction, impaired vascular reactivity, and chronic inflammation (6, 10, 11). This is particularly consequential because, unlike large-vessel disease, CSVD remains largely inaccessible to surgical or endovascular intervention, relying instead on medical therapy, risk factor control, and lifestyle modification. Advancing diagnostic precision and mechanistic understanding is therefore essential for identifying new preventive and therapeutic strategies.

Recent progress has been driven in large part by advances in neuroimaging. Modern MRI techniques now allow detailed assessment of the vessel wall, cerebral perfusion, vascular dynamics, and tissue microstructure, underscoring that cerebrovascular disease cannot be fully captured by static measures such as infarct volume or degree of stenosis. Instead, vascular pathology exerts its effects through dynamic processes that influence peri-lesional tissue, white matter integrity, and distributed brain networks. Imaging biomarkers sensitive to these processes are now reshaping how vascular brain injury and recovery are conceptualized (2, 12).

These imaging advances are exemplified by the work of Jiang et al., who synthesize evidence demonstrating that neuroimaging can capture biologically meaningful markers of brain repair following ischemic stroke, particularly within peri-infarct regions and distributed functional networks. Their findings underscore the limitations of infarct volume as a surrogate endpoint and point toward white matter integrity, functional reorganization, and perfusion as more sensitive indicators of recovery.

Taken together, these findings suggest that cerebrovascular disease involves disturbances in systems-level regulation in addition to focal injury (6, 11). Vascular dysfunction is associated with disrupted neurovascular coupling, impaired metabolic efficiency, and reduced neural plasticity. Functional and metabolic adaptations frequently extend beyond the primary site of injury, reflecting bilateral and network-level responses that shape long-term outcomes. Such observations are particularly relevant to CSVD, where diffuse microvascular injury accumulates over time and manifests clinically as cognitive decline, gait disturbance, and mood disorders rather than discrete neurological deficits (6, 11).

Quantifying CSVD burden remains challenging, and composite imaging scores, whether visually rated or computationally derived, have emerged as practical tools to capture its multifaceted nature (2, 6, 13). The meta-analysis by Silva et al. supports the reliability and construct validity of these scores, while also highlighting variability related to imaging protocols, rater characteristics, and population differences. This variability underscores the need to relate imaging phenotypes to underlying biological mechanisms, an effort that may benefit from integrating imaging with systemic biomarkers and longitudinal clinical data.

Mounting evidence suggests that cerebrovascular pathology reflects a close interplay between cerebral and systemic processes. Associations between peripheral inflammatory markers, white matter injury, and brain atrophy, as explored by Liu et al., support the concept that immune and inflammatory pathways actively modulate microvascular injury and tissue vulnerability. These findings align with a growing body of literature implicating chronic inflammation and endothelial dysfunction as drivers of both CSVD and vascular cognitive impairment (6).

Beyond perfusion and inflammation, new concepts emphasize the role of perivascular and clearance pathways in maintaining cerebral homeostasis. The framework advanced by Corbali and Levey positions the glymphatic system as a potential mediator linking vascular pulsatility, sleep physiology, and metabolic waste clearance. Impairment of these pathways may provide a mechanistic bridge between vascular dysfunction, protein accumulation, and neurodegeneration. A notable example is the association between cerebral amyloid angiopathy and Alzheimer's disease (14). Even as methodological challenges limit clinical translation, integrating perivascular dynamics into cerebrovascular research expands conceptual boundaries and identifies new avenues for intervention.

At the acute end of the spectrum, cerebrovascular disease also exhibits marked biological heterogeneity. This is further illustrated by Zhao et al., who show that elevated cardiac troponin levels are strongly associated with adverse neurological and cardiopulmonary outcomes in aneurysmal subarachnoid hemorrhage, highlighting the tight coupling between acute cerebrovascular injury, systemic stress responses, and clinical prognosis. From a therapeutic perspective, Lenhart et al. illustrate how targeted pharmacological strategies can be used to manage procedure-related thromboembolic complications during endovascular treatment, highlighting how evolving periprocedural therapies may mitigate risk and enhance technical success. Histopathological analyses of thrombus material, as reported by Rojas-Bartolomé et al., reveal complex clot architectures shaped by inflammatory and cellular components, with potential implications for reperfusion strategies and treatment resistance. Similarly, hemodynamic studies of large-vessel pathology, such as those by Lee et al., demonstrate that proximal arterial disease can induce downstream alterations in cerebral flow patterns and vascular stress, reinforcing the concept of the cerebrovascular system as an integrated network. Finally, Halvorsen et al. provide important insight into chronic large-vessel remodeling, showing that age-related stiffening of the anterior cerebral artery is associated with smooth muscle cell atrophy and disorganization of adventitial collagen, underscoring how structural vascular changes evolve over time and contribute to long-term cerebrovascular vulnerability.

Together, these findings emphasize that vascular diseases of the brain are dynamic, multifactorial disorders that benefit from integrative approaches (6, 10). Advances in artificial intelligence and machine learning offer new avenues for handling the complexity of multimodal data, enabling automated detection of subtle imaging features, improved risk stratification, and outcome prediction. When coupled with standardized imaging pipelines and biologically informed models, these approaches hold promise for translating scientific insight into clinical impact (1–3, 13).

In summary, recent advances have significantly enhanced the understanding of cerebrovascular disease, particularly CSVD, while also revealing persistent gaps in knowledge and available therapies. Moving beyond lesion-centric paradigms toward systems-based models that integrate vascular biology, neuroimaging, and computational tools will be essential for addressing the burden of vascular brain disease and mitigating its contribution to cognitive decline. Critical challenges remain, including the need for standardized imaging protocols across centers, validation of imaging biomarkers in diverse populations, and development of therapeutic interventions that target the mechanisms identified through advanced imaging.

## References

[1] Yao AD Cheng DL Pan I Kitamura F. Deep Learning in neuroradiology: a systematic review of current algorithms and approaches for the new wave of imaging technology. Radiol Artif Intell. (2020) 2:e190026. doi: 10.1148/ryai.2020190026 33937816 PMC8017426

[2] Duering M Biessels GJ Brodtmann A Chen C Cordonnier C de Leeuw FE . Neuroimaging standards for research into small vessel disease-advances since 2013. Lancet Neurol. (2023) 22:602–18. doi: 10.1016/S1474-4422(23)00131-X 37236211

[3] Cheng PM Montagnon E Yamashita R Pan I Cadrin-Chenevert A Perdigon Romero F . Deep learning: an update for Radiologists. Radiographics. (2021) 41:1427–45. doi: 10.1148/rg.2021200210 34469211

[4] GBD 2021 Stroke Risk Factor Collaborators. Global, regional, and national burden of stroke and its risk factors, 1990-2021: a systematic analysis for the Global Burden of Disease Study (2021). Lancet Neurol. (2024) 23:973–1003. doi: 10.1016/S1474-4422(24)00369-7 39304265 PMC12254192

[5] Wasselius J Arnberg F von Euler M Wester P Ullberg T. Endovascular thrombectomy for acute ischemic stroke. J Intern Med. (2022) 291:303–16. doi: 10.1111/joim.13425 35172028

[6] Wardlaw JM Smith C Dichgans M. Small vessel disease: mechanisms and clinical implications. Lancet Neurol. (2019) 18:684–96. doi: 10.1016/S1474-4422(19)30079-1 31097385

[7] Tjoumakaris SI Hanel R Mocco J Ali-Aziz Sultan M Froehler M Lieber BB . ARISE I consensus review on the management of intracranial aneurysms. Stroke. (2024) 55:1428–37. doi: 10.1161/STROKEAHA.123.046208 38648283

[8] Rehwald R. Improving the prediction of intracranial aneurysm rupture risk-calcification in the spotlight. Eur Radiol. (2024) 34:7514–6. doi: 10.1007/s00330-024-10990-3 39075301

[9] Pantoni L. Cerebral small vessel disease: from pathogenesis and clinical characteristics to therapeutic challenges. Lancet Neurol. (2010) 9:689–701. doi: 10.1016/S1474-4422(10)70104-6 20610345

[10] Brisset M Boutouyrie P Pico F Zhu Y Zureik M Schilling S . Large-vessel correlates of cerebral small-vessel disease. Neurology. (2013) 80:662–9. doi: 10.1212/WNL.0b013e318281ccc2 23345633 PMC3590057

[11] Markus HS de Leeuw FE. Cerebral small vessel disease: recent advances and future directions. Int J Stroke. (2023) 18:4–14. doi: 10.1177/17474930221144911 36575578 PMC9806465

[12] Paradise MB Shepherd CE Wen W Sachdev PS. Neuroimaging and neuropathology indices of cerebrovascular disease burden: a systematic review. Neurology. (2018) 91:310–20. doi: 10.1212/WNL.0000000000005997 30021917

[13] Lohner V Badhwar A Detcheverry FE Garcia CL Gellersen HM Khodakarami Z . Machine learning applications in vascular neuroimaging for the diagnosis and prognosis of cognitive impairment and dementia: a systematic review and meta-analysis. Alzheimers Res Ther. (2025) 17:183. doi: 10.1101/2024.12.17.24319166 40775365 PMC12330124

[14] Jager HR. The connection between cerebral amyloid angiopathy and Alzheimer's disease. Eur Radiol. (2024) 34:2171–3. doi: 10.1007/s00330-023-10462-0 38062269

